# A randomized double-blind placebo-controlled trial to investigate the effects of nasal calcitonin on bone microarchitecture measured by high-resolution peripheral quantitative computerized tomography in postmenopausal women — Study protocol

**DOI:** 10.1186/1745-6215-9-19

**Published:** 2008-04-13

**Authors:** Laura Richert, Brigitte Uebelhart, Marc Engelhardt, Moise Azria, René Rizzoli

**Affiliations:** 1 Service of Bone Diseases, Department of Rehabilitation and Geriatrics, University Hospitals of Geneva, 24, Rue Micheli-du-Crest, 1211 Geneva 14, Switzerland; 2 Novartis Pharma AG, 4002 Basel, Switzerland

## Abstract

**Background:**

Bone microarchitecture is a significant determinant of bone strength. So far, the assessment of bone microarchitecture has required bone biopsies, limiting its utilization in clinical practice to one single skeletal site. With the advance of high-resolution imaging techniques, non-invasive in vivo measurement of bone microarchitecture has recently become possible. This provides an opportunity to efficiently assess the effects of anti-osteoporotic therapies on bone microarchitecture. We therefore designed a protocol to investigate the effects of nasal salmon calcitonin, an inhibitor of osteoclast activity, on bone microarchitecture in postmenopausal women, comparing weight bearing and non-weight bearing skeletal sites.

**Methods:**

One hundred postmenopausal women will be included in a randomized, placebo-controlled, double-blind trial comparing the effect of nasal salmon calcitonin (200 UI/day) to placebo over two years. Bone microarchitecture at the distal radius and distal tibia will be determined yearly by high-resolution peripheral quantitative computerized tomography (p-QCT) with a voxel size of 82 μm and an irradiation of less than 5 μSv. Serum markers of bone resorption and bone formation will be measured every 6 months. Safety and compliance will be assessed. Primary endpoint is the change in bone microarchitecture; secondary endpoint is the change in markers of bone turnover.

**Hypothesis:**

The present study should provide new information on the mode of action of nasal calcitonin. We hypothezise that - compared to placebo - calcitonin impacts on microstructural parameters, with a possible difference between weight bearing and non-weight bearing bones.

**Trial Registration:**

ClinicalTrials.gov NCT00372099

## Background

Osteoporosis is characterized by compromised bone strength and higher fragility, resulting in an increased fracture risk [1]. The measurement of areal bone mineral density (BMD) by dual-energy X-ray absorptiometry (DXA) is the most common way to measure bone mass and to assess fracture risk. Yet, bone strength is also determined by bone geometry, bone microarchitecture, bone remodelling and intrinsic tissue quality [2]. Thus, it is not surprising that BMD only partially explains the variance of strength and fracture risk [3]. It has been postulated that markers of bone remodelling determined in blood or urine could be independent predictors of fracture risk [4]. Assessment of changes in bone microarchitecture has been limited, since bone biopsies were necessary to investigate these parameters in the past. This technique is limited to a non-weight bearing bone such as the iliac crest. Today, high-resolution peripheral quantitative computerized tomography (p-QCT) allows an efficient and quantitative evaluation of bone microarchitecture [5,6]. It determines in three dimensions true volumetric density (g/cm^3^), and can directly assess trabecular thicknesses, trabecular separations, and trabecular number, and also provides additional geometric variables such as cross-sectional area (CSA) and information on structural pattern (rod vs. plate structure) with a relatively low irradiation, high accuracy and precision [7]. P-QCT thus provides a unique non-invasive opportunity to explore the effects of diseases on bone microarchitecture and of therapies intended to halt or reverse these changes [8].

Postmenopausal bone loss and fracture risk are associated with an imbalance in bone turnover. As a therapeutic agent, calcitonin binds to specific receptors on the osteoclasts and reduces their relative hyperactivity.

The formula of nasal calcitonin is well tolerated. The most common side effects consist of mild or moderate rhinitis symptoms, such as nasal congestion and sneezing, or nasal dryness [9,10]. Rarely, hypersensitivity reactions may occur.

Prolonged administration of intranasal calcitonin can prevent postmenopausal bone loss and is also able to increase trabecular bone mass among patients presenting established osteoporosis [10,11]. It has been demonstrated that the prolonged daily administration of 200 IU intranasal calcitonin inhibits bone resorption, increases lumbar spine BMD by 1.7%–3.3% after one year, and reduces vertebral fracture risk [9,12]. Furthermore, specific modification of bone microstructure was demonstrated at the distal radius using magnetic resonance imaging [13].

A possible effect of calcitonin on bone microarchitecture could be investigated using high-resolution p-QCT. By assessing structural bone changes, the present study should thus provide new insights in the mode of action of nasal calcitonin. We hypothezise that - compared to placebo - calcitonin improves or maintains microstructural parameters, with a possible difference between weight bearing and non-weight bearing bones.

## Methods

### Study design

This is a monocentric, randomized, placebo-controlled, double-blind trial over two years, assessing the effects of nasal calcitonin (200 IU/day) on bone microarchitecture measured by high resolution p-QCT in postmenopausal women.

This protocol was initiated by the principal investigator and has been accepted by the ethics committee of the University Hospitals of Geneva and by the Swiss legislative authority (Swissmedic). It has been registered in a public clinical trial data base [ClinicalTrials.gov number NCT00372099] [14].

### End points

Primary efficacy endpoint will be changes in bone structure and bone microarchitecture evaluated by high resolution p-QCT in postmenopausal women compared to placebo during two years.

As a secondary endpoint we will investigate changes in markers of bone turnover.

### Study population

The study population will consist of 100 postmenopausal women aged 45 to 70 years with a BMD T-Score between 0.0 and -2.49. In- and exclusion criteria are detailed in Table 1. Given the possible administration of placebo treatment, women with osteoporosis are excluded from the study for ethical reasons. Study participants are being recruited through advertisements in local newspapers; recruitment has started in January 2007.

**Table 1:** In- and exclusion criteria

**Inclusion criteria**
• Women aged 45 to70 years
• Natural or surgical menopause = 1 year
• T-Score between 0.0 and -2.49 (spine or proximal femur – total or femoral neck, measured by DXA)

**Exclusion criteria**

• Osteoporosis (T-score = -2.5 in spine or proximal femur – total or femoral neck, measured by DXA)
• Severe vertebral fracture, as identified by screening DXA assessment
• Any history of metabolic disease that could affect bone metabolism: hyperparathyroidism, osteogenesis imperfecta, Paget's disease, osteomalacia
• Thyroid disease (if receiving thyroid hormone replacement, the patients must be euthyroid and on a stable dose of thyroid hormone)
• Impaired renal function (estimated creatinine clearance <30 ml/min)
• History of previous or active malignancy of any organ system, treated or untreated, within the past 5 years.
• History of corticosteroid treatments during 6 months or more, daily dosage >5 mg
• BMI < 18 or >30 kg/m^2^
• Treatments with estrogens, SERMs, tibolone, calcitonin, strontium ranelate, teriparatide or PTH, or oral bisphosphonates in the previous year, if duration > 2 weeks. Treatment with iv bisphosphonates or iv calcitonin of any duration in the previous year

### Study medication

Participants receive 200 IU calcitonin nasal spray once daily or the matching placebo spray, provided by Novartis Pharmaceuticals, Basel, Switzerland. All patients fulfilling the inclusion/exclusion criteria will be given study medication. Odds of receiving active treatment or placebo are 1:1. Medication labels comply with the legal requirements in Switzerland and will be printed in the local language. The storage conditions for the study drug as well as the randomization number are specified on the medication label. No patient identifier is stated on the label.

All patients receive a calcium and vitamin D supplement (1000 mg/880 UI per day). If the supplement is not tolerated, the dose will be reduced or, if necessary, the supplement will be stopped. In this case, the participant may continue the trial and will be given advice on adequate dietary calcium intake. A vitamin D supplement of 800 IU/day will then be prescribed during the winter months.

Drug accountability will be noted during visits and at the completion of the trial. Patients will be asked to return all unused medication at the end of the study.

### Blinding

Patients, investigator staff, persons performing the assessments, and data analysts will remain blind to the identity of the treatment from the time of randomization until database lock. The identity of the treatments will be concealed by the use of study drugs that are all identical in packaging, labeling, schedule of administration, appearance, taste, and odor. Randomization data are kept strictly confidential until the time of unblinding and will not be accessible by anyone else involved in the study. Unblinding will only occur in the case of patient emergencies and at the conclusion of the study.

### Study procedures

Potential participants will undergo a screening procedure, including recording of medical history, physical examination, blood sampling, and BMD measurements and vertebral fracture assessment (VFA) by DXA (Prodigy, GE Healthcare Technologies, Waltham, Wisconsin, USA). Participants fulfilling the inclusion/exclusion criteria at screening will be included in the study. During the study, visits to the trial center will take place every 6 months (Table 2).

**Table 2:** Visit schedule

	Visit 1	Visit 2	Visit 3	Visit 4	Visit 5	Visit 6
Period	Screening	Baseline	Month 6	Month 12	Month 18	Month 24

Day	-7 to -3	0	180 ± 14	360 ± 14	540 ± 14	720 ± 14

Informed consent	X					
In-/exclusion criteria	X	X				
Medical history	X	X				
Prior/Concomitant medication	X	X	X	X	X	X
Vital signs		X		X		X
Physical examination	X					X
Adverse events			X	X	X	X
DXA	X			X		X
pQCT		X		X		X
Laboratory test	X			X		X
Bone markers	X	X	X	X	X	X
Antibody test						X
Study drug dispensing		X	X	X	X	
Drug accountability			X	X	X	X
Study termination sheet						X

### Efficacy assessments

#### Bone microarchictecture measurements

Bone microarchitecture will be investigated by p-QCT at baseline and after one and two years. Three-dimensional high-resolution measurements will be performed at the distal radius and tibia using an Xtreme CT device (Scanco Medical AG, Bassersdorf, Switzerland), as described by others [15]. In brief, the non-dominant limb (or the nonfractured limb in case of prior fractures) will be scanned at the standardized locations for human in vivo measurements. The following settings are used: effective energy of 60 kVp, matrix size 1536 × 1536, x-ray tube current 900 μA, slice increment 82 μm. One hundred and ten slices will be obtained with a voxel size of 82 μm. The effective dose for one Xtreme CT standard measurement is 3 μSv per site. Processing of the obtained scans will be done in accordance with the default protocol of the device. Table 3 lists the assessed bone parameters. In our institution, the in-vivo coefficient of variation (CV%) for these bone indices ranges from 0.5 – 1% (vol. densities) to 3 – 5% (TbN). Quality control is performed on a daily basis.

**Table 3:** Parameters of bone microarchitecture

**Bone densities**
• cortical bone density (Dcort; mgHA/cm^3^)
• trabecular bone density (Dtrab; mgHA/cm^3^)
• meta trabecular bone density (Dmeta; mgHA/cm^3^)
• inner trabecular bone density (Dinn; mgHA/cm^3^)
• average bone density (Dtot; mgHA/cm^3^)

**Bone structure**

• trabecular bone volume to tissue volume (BV/TV; %)
• number of trabeculae (Tb.N; mm^-1^)
• trabecular thickness (Tb.Th; *μ*m)
• trabecular separation (Tb. Sp; *μ*m)
• cortical thickness (C.Th; *μ*m)
• index of the inhomogeneity of the network (Tb.1/N.SD)
• cross-sectional area (CSA; mm^2^)

#### Bone mineral density measurements

Spine and hip bone mineral density will be measured at the baseline visit and after one and two years by DXA (Prodigy, GE Healthcare Technologies, Wisconsin, USA). The irradiation dose is 7 μSv per examination.

#### Laboratory test

Fasting serum samples will be obtained from all patients at the 6-monthly visits. Serum will be stored at -70°C to allow analysis in batches. Serum total procollagen type 1 amino-terminal propeptide (P1NP), a marker of bone formation, and serum C-terminal telopeptide of type I collagen (β-crosslaps, CTX), a marker of bone resorption, will both be measured using electrochemiluminescence immunoassays (Elecsys 2010, Roche Diagnostics). The intrassay coefficients of variation (CV%) for these measurements are 2.3–3.7% and 1.6–4.7%, respectively. Levels of serum PTH (7–84) will also be assessed using an electrochemiluminescence immunoassay (CV 4.3–7.1%); serum 25-hydroxyvitamin D will be determined by a competitive immunoassay (ELISA; CV 5.3–6.7%).

Serum and urine biochemistry as well as hematology will be measured immediately after blood sampling in the routine laboratory according to standard procedures. The titer of calcitonin antibodies will be assessed at end of the study.

### Safety assessments

Safety assessments will include the monitoring and recording of all adverse events (AEs), including serious adverse events (SAEs), the regular monitoring of hematology, blood chemistry and urine values, regular monitoring of vital signs, physical condition and body weight. Body height will be measured 6-monthly with a Harpeden stadiometer (Holtain Ltd, Crymych, UK).

### Data management

All original data will be recorded in the patient's source documentation. Subsequently, anonymized data will be entered into an electronic database and displayed in the patient data listings. Tables will display counts of missing values. Quality control of the data will be done at regular intervals, using descriptive statistics and visual analyses to check for inconsistencies and extreme values. Detected errors will be rectified according to the source data. Data will be handled in accordance with the good clincal practice guidelines and applicable local regulations. Data management and analysis as well as interpretation and publication of the results remain under the responsibility of the principal investigator and will be performed by the study team of the University Hospitals of Geneva.

### Statistical methods

#### Sample size calculation

A total number of 80 patients (40 in the calcitonin group and 40 in the control group) is necessary to have a statistical power of 80% to detect an expected difference of 3.5% in BV/TV [13]. Due to an expected drop-out rate of 20%, a total of 100 patients should be enrolled.

#### Statistical analysis

Data will be summarized with respect to demographic and baseline characteristics, efficacy observations and measurements, and safety observations and measurements.

To assess the treatment effects of calcitonin on bone microarchitecture and provide a proof of concept, the primary analysis of the efficacy endpoints in this exploratory trial will be based on the observed data of the per-protocol population. Secondarily, an ITT analysis may be performed to test the robustness of the results.

For each analyzed parameter a two-sided t-test will be applied. The change from baseline between the two different groups (active treatment/placebo) will be compared by an analysis of covariance (ANCOVA) model with change as the response variable, the baseline value as a covariate, and treatment as a fixed factor.

The assessment of safety will be based mainly on the frequency of adverse events and on the number of laboratory values that fall outside of pre-determined ranges. Other safety data (e.g. electrocardiogram, vital signs, special tests) will be considered as appropriate.

Analysis of adverse events will be based on the safety population which includes all randomized patients who have taken at least one dose of the study drug. Adverse events will be summarized by presenting, for each treatment group, the number and percentage of patients having any adverse event, having an adverse event in each body system and having each individual adverse event. Any other information collected (e.g. severity or relatedness to study medication) will be listed as appropriate.

## Summary

This study will investigate the effects of nasal salmon calcitonin on bone microarchitecture and its relation with markers of bone turnover in postmenopausal women. It should thus give a new insight into the specific effects of calcitonin on different bone parameters.

## Competing interests

M.E. and M.A. are employees of Novartis. All other authors declare that they have no competing interests. This study is financially supported by Novartis.

## Authors' contributions

LR participated in the design and coordination of the study, was responsible for the data acquisition and management, and drafted the manuscript. BU contributed to study coordination and clinical care of the trial participants. ME and MA provided useful advice for the study design. RR is the initiator and principal investigator of the study and drafted the original protocol. All authors read and approved the final manuscript.
